# Probiotics in Treatment of Allergic Rhinitis

**DOI:** 10.1097/WOX.0b013e3181f234d4

**Published:** 2010-09-15

**Authors:** Rashmi Ranjan Das, Meenu Singh, Nusrat Shafiq

**Affiliations:** 1 Department of Pediatrics, All India Institute of Medical Sciences (AIIMS), New Delhi, India; 2 Department of Pediatrics, Advanced Pediatrics Centre (APC), Post-Graduate Institute of Medical Education and Research (PGIMER), Chandigarh, India; 3 Department of Pharmacology, Post-Graduate Institute of Medical Education and Research (PGIMER), Chandigarh, India

**Keywords:** respiratory allergy, lactobacillus, quality of life, biofidobacterium, clinical trials

## Abstract

Many randomized controlled trials (RCTs) have been done on role of probiotics as a treatment modality in allergic rhinitis. We conducted a review on the same. A systematic search of published literature was done. RCTs comparing effect of probiotics with placebo were included. A predefined set of outcome measures were assessed. Continuous data were expressed as pooled standardized mean difference (SMD) with 95% confidence interval (CI). Dichotomous data were expressed as odds ratio with 95% CI. *P *value < 0.05 was considered significant. RevMan version 5 was used for all the analyses. Seven RCTs were eligible for inclusion. Probiotic intake improved quality of life score in patients with allergic rhinitis [SMD -1.17 (95% CI -1.47, -0.86; *P *< 0.00001)]. Other parameter that improved with probiotic intake was decrease in the number of episodes of rhinitis per year. There was no significant change in blood or immunologic parameters in the probiotic group, SMD -0.10 (95% CI -0.26, 0.06; *P *= 0.22). Adverse events were not significant. Probiotic therapy might be useful in rhinitis, but the present data do not allow any treatment recommendations.

## Introduction

In the past 4 decades, there has been a marked increase in the prevalence of allergic rhinitis (AR) in urban settings and a smaller rise in rural areas. In prosperous societies, 20-40% of children suffer from AR [1]. There is currently no cure for, though a wide range of treatments are employed to control the symptoms. Many of the treatment modalities do not act through modification of inflammatory pathways which underlies the pathophygiologic basis of these diseases.

Probiotics are live micro-organisms that confer a beneficial physiological effect on the host when administered in adequate amounts [2]. Limited evidence from systematic reviews shows that probiotics are beneficial for treating infectious diarrhea, preventing antibiotic-associated diarrhea, and treating vaginal infections in pregnancy [3-5]. They have been formally investigated in a number of clinical trials for the treatment of seasonal and perennial AR with variable results. The aim of the present paper is to find whether probiotics are effective in treatment of AR or not.

## Method

### Criteria for Considering Studies for This Review

#### Types of Studies

Randomized double-blind placebo-controlled trials (RCTs).

#### Types of Participants

Participants in trials were of either sex and of any age.

#### Types of Interventions

Interventions consisted of daily treatment with probiotics or placebo (no probiotic bacteria), used for > 2 weeks, as an additive to standard antiallergic medications. All formulations of probiotics (irrespective of the type, strain, and concentration) were considered.

#### Types of Outcome Measures

A. Primary outcome

Quality of life score at the end of treatment

B. Secondary outcomes

Time (months) free from episodes.

Mean duration of an episode.

Number of episodes per year.

Changes in blood parameters/immunologic markers.

Time or duration was defined was defined as number of days to resolution of specific outcome from initiation of treatment. Change in symptom score was defined as the change in total score over days per week. If the data were not available in the required format, the authors were contacted for the information.

### Search Methods for Identification of Studies

We systematically searched Medline, Cochrane Central Register of Controlled Trials (CENTRAL), EMBASE and previous reviews including cross references (all articles referenced), abstracts, and conference proceedings for all relevant articles till June 2010. Following keywords: "probiotics" OR "lactobacillus" OR "bifidobacterium" OR "bacterio-therapy" OR "fermented milk" OR "lactic acid bacteria" AND "supplement" OR "treatment" AND "allergy" OR "respiratory allergy" OR "allergic rhinitis" AND "children" OR "pediatric" OR "adults" AND "clinical trial" OR "randomized controlled trial" were used for retrieval of relevant articles. No language restrictions were applied. Two investigators carried out the search independently. We then combined all the searches and retrieved the relevant articles. Manual search was made by going through the reference lists of the retrieved articles and through Index Medicus and key allergy, asthma, and immunology journals.

### Data Collection and Analysis

#### Methodological Quality

Each included study was evaluated with the (previously validated) 5-point Jadad scale to assess quality of trials by 2 independent reviewers [6]. This scale assigns points as follows:

1. Was the study described as randomized? (0 = no; 1 = yes)

2. Was the study described as double-blind? (0 = no; 1 = yes)

3. Was there a description of withdrawals and drop-outs? (0 = no; 1 = yes)

4. Was the method of randomization well described and appropriate? (0 = no; 1 = yes)

5. Was the method of double blinding well described and appropriate? (0 = no; 1 = yes)

6. Deduct 1 point if methods for randomization or blinding were inappropriate.

Out of maximum possible score of 5, studies with scores ≥ 3 are supposed to be of good qualities were included in the analysis.

#### Data Collection

Two review authors independently reviewed the results for inclusion in the analysis. Design of the trial, comparator, characteristics of study participants, number of participants, type of intervention (dose, duration), and major outcomes evaluated. Differences about study quality were resolved through discussion. We recorded data on a prestructured data extraction form. We assessed publication bias using the Cochrane Collaboration's 'Risk of bias' tool.

#### Study Descriptions

Information in relation to methodological quality, characteristics of participants, interventions and outcome measures of each trial is provided in Table 1.

**Table 1:** Characteristics of Included Studies

Study/Year	Participants	Intervention	Outcomes	Notes	Jadad Score
Peng 2005[7]	1) 90 subjects (probiotics = 60, placebo = 30) in 3 groups 2) Age (yrs) 16.07 ± 2.11 (live), 14.50 ± 1.78 (heat-killed), 16.60 ± 2.02 (placebo) 3) Allergic rhinitis symptoms for > 1 year and sensitization to house dust mites	1) *Lactobacillus paracasei *(5 × 10^9 ^cfu/capsule) 2) 2 capsules/d of live, heat killed, and placebo capsules in 3 groups 3) Total duration = 30 days	Primary: change in symptom scores on the modified PRQLQ from baseline following treatment	No drop-out from study	3
Wang 2004[8]	1) 80 children (probiotics = 60, placebo = 20) 2) Age (yrs) 15.87 ± 1.53 (intervention) and 14.00 ± 1.90 (placebo) 3) Allergic rhinitis symptoms for > 1 year and sensitization to house dust mites	1) *Lactobacillus paracasei*-33 (2 × 10^9 ^cfu/200 ml) 2) 200-400 mL/d 3) Total duration = 30 days	Primary: the change in symptom scores of the modified PRQLQ	No drop-out from study	3
Giovannini 2007[9]	1) 187 children (probiotics = 92, placebo = 95) 2) Age 2-5 years 3) Global Initiative for Asthma (GINA) and Allergic Rhinitis and its guidelines used	1) *Lactobacillus bulgaricus*, 10^7^cfu/ml, *Streptococcus thermophilus*, 10^8 ^cfu/ml and *L. casei *10^8 ^cfu/ml 2) 100 mL/d 3) Total duration = 12 months	Number of days free from episodes of asthma and/or rhinitis, and cumulative number and duration of episodes, change in immunologic profile	Participants were using standard asthma and allergic medication, 29 patients LFU	5
Tamura 2007[10]	1) 120 subjects (probiotics = 60, placebo = 60) 2) Age range (yr); intervention (39.3 ± 8.0) and placebo (39.5 ± 10.9) 3) Having specific IgE for JCP and symptomatology defined by the Japanese society of Allergology	1) *Lactobacillus casei shirota *(4 × 10^10 ^cfu/80 mL) 2) 80 mL/d 3) Total duration = 8 weeks	Change in SMS, immunological profile and any side-effect noted	Standard allergy medications used by subjects, 11 patients LFU	4
Xiao 2006a[11]	1) 40 adult subjects (probiotics = 20, placebo = 20) 2) Age range; 23-61 years (intervention) and 24-55 years (placebo) 3) More than 2-year clinical history of JCPsis and presence of anti-JCP-IgE	1) *Bifidobacterium longum*536 (BB536); 3.5 ± 2.4 × 10^8 ^cfu/100 g 2) 200 g/d 3) Total duration = 14 weeks	Effect on subjective symptoms score, any effect on blood parameters	Standard care for allergic symptoms	4
Xiao 2006b[12]	1) 44 adult subjects (probiotics = 22, (probiotics = 22, placebo = 22) 2) Age range; 22 to 48 years (placebo) and 26 to 57 years (intervention) 3) 2-year clinical history of JCPsis and the presence of anti-JCP-IgE	1) BB536 powder 5 × 10^10 ^cfu/2 g 2) Twice daily 3) Total duration = 13 weeks	Effect on subjective symptom scores and blood parameters	Standard care for allergic symptoms. 11 patients LFU	4
Ishida 2005[13]	1) 49 adult subjects (probiotics = 25, plaebo = 24) 2) Age range was 34.0 ± 3.4 years (intervention) and 36.9 ± 3.0 yrs (placebo) 3) Allergic rhinitis (defined by the Japanese Society of Allergology) and high IgE antibody against house dust or house dust mite	1) *Lactobacillus Acidophilus *L-92 (3 × 10^10 ^cfu/100 mL) 2) 100 mL/d 3) Total duration = 8 weeks	Change in SMS (both nasal and ocular) value. Any change in nasal cavity findings and blood parameters	Standard allergy medications used, 3 patients LFU	3

RCT, Randomized double-blind placebo controlled trials; FU, follow-up, cfu, colony forming unit; SMS, symptom-medication score; JCP, Japanese cedar pollinosis; PRQLQ, Pediatric Rhinoconjunctivitis Quality of Life Questionnaire; LFU, lost to follow up.

#### Data Synthesis

Continuous data were expressed as mean (SD) and standardized mean difference (SMD) was obtained. The data from various studies were pooled and expressed as pooled SMD with 95% confidence interval (CI). Dichotomous data were expressed as odds ratio (OR) with 95% CI. *P *value < 0.05 was considered significant. A fixed effects model was initially conducted. If significant heterogeneity existed between trials, potential sources of heterogeneity were considered and where appropriate a random effects model was used. Inverted funnel plot was generated for assessment of publication bias. RevMan (Version 5) was used for all the analyses.

## Results

**Figure 1:**
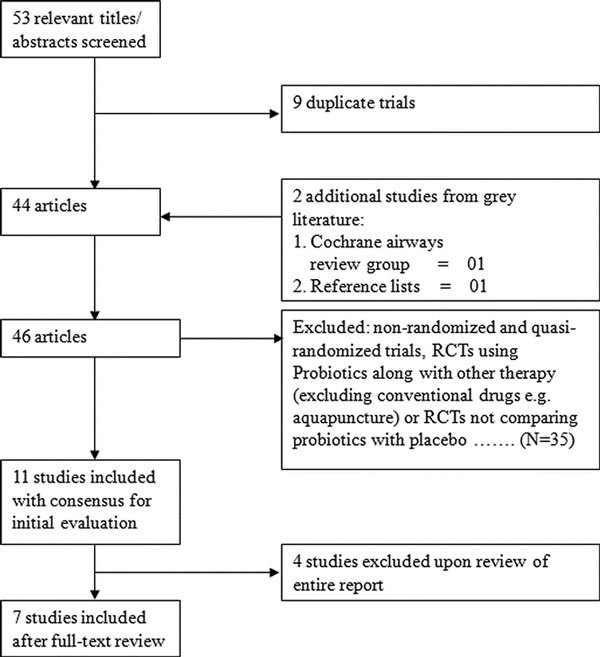
**Flow diagram of search results**. RCTs = Randomized controlled trials.

There were 53 hits obtained when the combined MeSH terms were used (Figure 1). From the initial search, 11 studies were considered as potentially eligible. These studies were further evaluated for eligibility. Seven studies were found to be eligible for inclusion in this review (Table 1),[7-13]and 4 studies were excluded [20-23]. The quality of studies were good with Jadad score varying from 3 to 5. Though most studies had adequate randomization and blinded intervention, allocation concealment was not clear in all but one study [9]. Not all studies assessed all the out comes. For the studies in which the results were expressed as mean (95% CI) or mean ± SE, the SD was derived from the available data. Seven included studies enrolled a total of 616 participants (342 for treatment and 274 as control subjects, which totaled 610 after losses to follow-up) involving all age groups and both sexes. In 3 trials, participants were administered probiotics on/before the onset of pollen season and was continued until the completion of the pollen season [10-12]. Two studies provided data on the assessment of quality of life[7,8]and 3 about adverse-events [7,8,10].

### Primary Outcome Measure (Figure 2)

#### Quality of Life Score at the End of Treatment

Two studies evaluated the quality of life score (frequency, level of bother) in 170 patients [7,8]. Compared with the placebo group, intervention group showed an improvement in the individual [change in frequency, SMD -0.90 (95% CI -1.34, -0.45; *P *< 0.00001) and change in level of bother, SMD -1.40 (95% CI -1.82, -0.98; *P *< 0.00001)], and overall quality of life score, SMD -1.17 (95% CI -1.47, -0.86; *P *< 0.00001).

**Figure 2:**
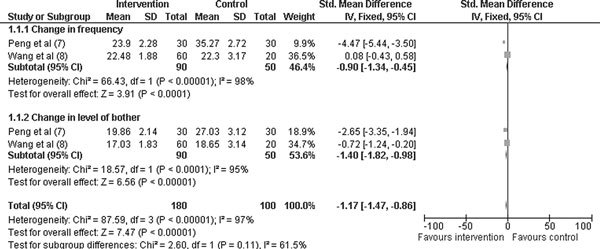
**Primary outcome measures (change in quality of life score)**.

### Secondary Outcome Measures

The results could not be pooled (except for blood/immunologic parameters) as there were single study reports.

#### Time (Months) Free From the Episodes

Probiotic intake has no effect on the time free from episodes of rhinitis. The mean (95% CI) time free from episodes being 4.1 (3.1 to 5.0) months in the intervention group versus 3.3 (2.4 to 4.3) months in the control group (*P *value = 0.9).

#### Mean Duration of an Episode

There was no significant difference between intervention and control group, [mean 1.02 (95% CI -0.27 to 2.32)].

#### Number of Episodes Per Year

The episodes of rhinitis were significantly lower in the intervention group with an adjusted odds ratio (95% CI) of 0.39 (0.19 to 0.82, *P *< 0.01).

#### Changes in Blood or Immunologic Parameters

Data from 5 studies including 372 patients were used for this analysis [9-13]. Overall there was no significant change in blood or immunologic parameters in the probiotic group, SMD -0.10 (95% CI -0.26, 0.06; *P *= 0.22).

#### Side-Effects Noted (If Any)

None of the 7 studies reported a definition of what constituted an adverse event. Two of the 3 studies, that did monitor for adverse events reported absence of adverse events [7,8]. The third, reported 14 minor adverse events (including cold, diarrhea, vomiting) but not the group (treatment or control) in which they occurred [10].

#### Publication Bias

**Figure 3:**
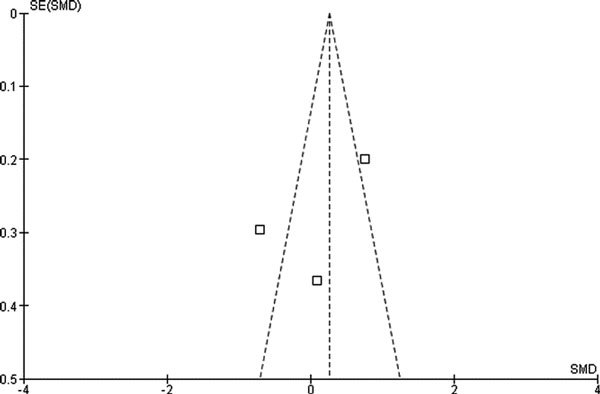
**Funnel plot**. Assessing publication bias using the SMD and 1/SE of SMD values from serum total IgE level.

To assess whether there was a bias in the published literature, funnel plot was constructed using the SMD and 1/SE (standard error of mean) of SMD values obtained from studies for one of the secondary outcome measures (serum total IgE level) as there were paucity of data for primary outcome measures. In the absence of a publication bias, such a plot is expected to have a shape resembling an inverted funnel [14]. From the funnel plot generated, the possibility of publication bias in the analysis could not be ruled out (Figure 3).

## Discussion

In the present review, treatment with probiotic was shown to improve the quality of life score of patients with AR at the end of treatment. Other outcome showing improvement with probiotic treatment was decrease in the number of episodes of rhinitis per year. Pooling could not be undertaken for many of the outcomes, as studies did not follow a standard format for reporting of clinical trials. Many reasons could account for the different outcomes among studies wherever pooling was done in these trials and these are: varied dose and intake period, the type and severity of the symptoms involved were different, seasonal variation of allergic symptoms and most importantly, the species and strains of the probiotics differ.

As atopic disease have seasonal variation of symptoms, the results could have been affected by time period of a particular study. Three studies reported effects of probiotics on allergic symptoms induced during pollen season of Japanese cedar pollen (JCP) in patients with history of such allergy (confirmed by symptoms and laboratory tests) [10-12]. In these trials, participants were administered probiotics on/before the onset of pollen season and were continued until the completion of the pollen season. BB536-supplemented yogurt has been demonstrated to have a pronounced promoting effect on intestinal environments after 2 weeks of intake at a dose of 100 g per day [15]. For this reason, in these studies probiotics was administrated before pollen exposure. Another important reason is the difference regarding the validity of the clinical effects of lactic acid bacteria among species and strains. In fact, in vitro studies using human mononuclear cells have indicated that there are strain-dependent differences in the ability of lactic acid bacteria to induce immunoregulatory monokines such as interleukin 12 [16]. Contribution of the species- and strain-specific nature of lactic acid bacteria on the efficacy of improving allergic symptoms should be considered.

Placebo was poorly defined in most of the studies. Many studies used nonfermented milk or plain yogurt as placebo. A better control would have been fermented milk without the addition of the probiotic bacteria or sterilized fermented milk [17]. The studies demonstrated the effect of fermented milk containing a specific probiotic strain, but it is not possible to conclude about the effect of probiotic bacteria per se. Indeed, studies state that plain yoghurt has some antiallergic effect and may have impact on rhinitis and asthma [18,19]. All these trials have used different doses and durations and different strains of probiotics (eg, bifidobacterium longum; lactobacillus strains). In all the trials, the minimum dose of probiotics administered was > 5 billion colony forming unit (CFU) and minimum duration of administration was 1 month. It has been hypothesized that some probiotic strains and/or their fermentation products are responsible for improvement of allergic rhinitis and the immunostimulatory effect of Lactobacillus may be dose dependant [11,15,20,21].

The effects of probiotics to modulate blood/immunologic parameters associated with allergic symptoms should be elucidated as some studies found beneficial effect on clinical parameters without significant change in the immunologic parameters. In this review, we found no significant overall change in immunologic parameters in the probiotics group. In all the trials, subjects were advised to continue antiallergic medications during symptomatic period. In contrast to other treatments such as histamine release inhibitors or antihistamines, the effects of probiotics are expected to be mild, with a lag period in the expression of their effect. Uses of medications vary from patient to patient and some has carried over effects (eg, steroids). Caution should be exercised during interpretation of results because of probiotic bacteria effects per se.

It is well known that systematic reviews are associated with limitations, and the results obtained with these methods should be analyzed accordingly. The numbers of patients analyzed were small to reflect the data on the whole population. Seven RCTs included a total of 616 subjects of both age and sex with a paucity of clinically relevant outcome measures. There was no uniformity in the definition of AR, and methodology of conducted trials. Indeed, this meta-analysis highlights the paucity of good quality clinical trials evaluating the role of probiotics in treatment of subjects with AR. To conclude, though probiotic therapy might be useful in rhinitis, the present data do not allow any treatment recommendations.

**Table 2:** Characteristics of Excluded Studies

Study	Reason for Exclusion
Trapp 1993[20]	Group 1 and 2 randomized and double-blinded, but group 3 were those did not want to eat yogurt
Helin 2002[21]	Patients had birch pollen allergy and mainly rhino-conjunctivitis
Xiao 2007[22]	Investigates symptoms induced by Japanese cedar pollen in an environmental exposure unit
Ishida 2005[23]	Randomized single-blind study with quality score = 0
